# Pathogenesis of virulent and attenuated foot-and-mouth disease virus in cattle

**DOI:** 10.1186/s12985-017-0758-9

**Published:** 2017-05-02

**Authors:** Jonathan Arzt, Juan M. Pacheco, Carolina Stenfeldt, Luis L. Rodriguez

**Affiliations:** 1 Foreign Animal Disease Research Unit, Plum Island Animal Disease Center, Agricultural Research Service, United States Department of Agriculture, Greenport, NY USA; 2 Oak Ridge Institute for Science and Education, PIADC Research Participation Program, Oak Ridge, TN USA

**Keywords:** Bovine, Cattle, FMD, FMDV, Foot-and-mouth, Pathogenesis, Virulence, Virus

## Abstract

**Background:**

Understanding the mechanisms of attenuation and virulence of foot-and-mouth disease virus (FMDV) in the natural host species is critical for development of next-generation countermeasures such as live-attenuated vaccines. Functional genomics analyses of FMDV have identified few virulence factors of which the leader proteinase (L_pro_) is the most thoroughly investigated. Previous work from our laboratory has characterized host factors in cattle inoculated with virulent FMDV and attenuated mutant strains with transposon insertions within L_pro_.

**Methods:**

In the current study, the characteristics defining virulence of FMDV in cattle were further investigated by comparing the pathogenesis of a mutant, attenuated strain (FMDV-Mut) to the parental, virulent virus from which the mutant was derived (FMDV-WT). The only difference between the two viruses was an insertion mutation in the inter-AUG region of the leader proteinase of FMDV-Mut. All cattle were infected by simulated-natural, aerosol inoculation.

**Results:**

Both viruses were demonstrated to establish primary infection in the nasopharyngeal mucosa with subsequent dissemination to the lungs. Immunomicroscopic localization of FMDV antigens indicated that both viruses infected superficial epithelial cells of the nasopharynx and lungs. The critical differences between the two viruses were a more rapid establishment of infection by FMDV-WT and quantitatively greater virus loads in secretions and infected tissues compared to FMDV-Mut. The slower replicating FMDV-Mut established a subclinical infection that was limited to respiratory epithelial sites, whereas the faster replication of FMDV-WT facilitated establishment of viremia, systemic dissemination of infection, and clinical disease.

**Conclusion:**

The mutant FMDV was capable of achieving all the same early pathogenesis landmarks as FMDV-WT, but was unable to establish systemic infection. The precise mechanism of attenuation remains undetermined; but current data suggests that the impaired replication of the mutant is more responsible for attenuation than differences in host immunological factors. These results complement previous studies by providing data of high-granularity describing tissue-specific tropism of FMDV and by demonstrating microscopic localization of virulent and attenuated clones of the same field-strain FMDV.

## Background

The continuum of attenuation and virulence of foot and mouth disease virus (FMDV; genus *Aphthovirus* family *Picornaviridae*) has been investigated in several studies, and multiple molecular determinants of virulence have been characterized [1–8]. However, the critical mechanisms which define the establishment of systemic disease remain incompletely elucidated. Understanding these mechanisms is important as improved knowledge of the functional genomics of FMDV may contribute towards improved FMD control and eradication through development of next-generation countermeasures such as live-attenuated vaccines.

Several studies have characterized clinical and pathological aspects of FMDV infection in cattle [9–14]. These works have indicated that early FMD pathogenesis in cattle involves critical events in the nasopharynx [11, 13], lungs [1, 10], or both [9, 12]. In recent years there have been several breakthroughs elucidating distinct functions of different FMDV-encoded proteins [7, 15, 16]; yet, the FMDV leader proteinase, (L_pro_). remains the most thoroughly investigated determinant of virulence (reviewed in [17]).

Virus constructs lacking the L_pro_ sequence (leaderless FMDV) have been shown to be avirulent in cattle and pigs [1, 18, 19]. Additionally, FMDV mutants with in-frame insertions in the inter-AUG spacer region of L_pro_ were shown to be markedly attenuated in cattle [3], but with improved replication dynamics compared to the leaderless mutants [20]. Recent works have demonstrated in vivo attenuation of various mutagenized FMDVs. These investigations have included viruses with deletions within the 3A coding region [6] or the VP1 GH-loop [5] as well as variable deletions or substitutions within the L_pro_ region [20, 21]. However, only two studies have directly compared the tissue-specificity of early pathogenesis events between a mutant FMDV and the virulent parental virus during the early stages of infection [1, 2].

Recent investigations from our laboratory have sought to elucidate mechanisms associated with host response to virulent and mutant-attenuated strains of FMDV-A24-Cruzeiro in cattle [2]. The overall conclusion based upon macroscopic analyses, was that the mutant virus was capable of establishing infection at similar primary sites as the parental virus; however, infection with the mutant was arrested at the primary infection sites by incompletely elucidated mechanisms which prevented dissemination and systemic disease. In the current work, we compared the early pathogenesis of the same strains of FMDV in larger cohorts of cattle by temporo-anatomic mapping of virus dynamics and immunomicroscopy in numerous tissues and secretions. The study herein provides extensive detail of the critical stages during early infection of cattle with FMDV. The time-specific anatomic distribution of virulent and attenuated strains of FMDV are described at whole-tissue and microanatomic levels. Microscopic localization of FMDV demonstrates infection of nasopharyngeal and pulmonary epithelial cells by both viruses. In the context of previously published findings, the current study further indicates that this FMDV-Mut was capable of achieving most of the critical molecular and cellular landmarks associated with infection and virulence, including cell entry and replication. The attenuated phenotype was seemingly determined by impaired replication dynamics which precluded dissemination and virulence.

## Methods

### Experimental animals

Twenty-nine 9–12 months old Holstein steers, weighing 300–450 kg were obtained from an experimental-livestock provider (Thomas-Morris Inc., Reisterstown, MD). Animals were housed individually in single-animal containment units within a BSL-3Ag animal facility from the time of inoculation until the time of euthanasia.

### Viruses

The virulent “wild type” FMDV utilized herein (FMDV-WT) was an infectious clone virus of a field isolate of FMDV- A_24_-Cruzeiro [22]; the mutant attenuated strain (FMDV-Mut) was derived from FMDV-WT by transposon insertion mutagenesis as previously described [2, 3]. Briefly, FMDV-Mut contains a random, 57 nucleotide, in-frame insertion in the region located between the two functional initiation codons (inter-AUG), within the leader proteinase (L_pro_). Virus inoculum for aerosol inoculations consisted of 10^7^ TCID_50_ FMDV in 2.0 ml of Minimum Essential Media (Gibco, San Diego, CA) with 25 mM Hepes.

### Experimental design

Twenty steers were infected with FMDV-WT and nine with FMDV-Mut by aerosol inoculation as previously described [9, 12]. Each steer was sedated with xylazine and fitted with a commercially available aerosol delivery system (Aeromask-ES, Trudell Medical, London, Ontario, Canada) which was placed over the muzzle. The mask was attached to a jet nebulizer (Whisper Jet, Vital Signs Inc., Totowa, NJ) which was subsequently attached to an air compressor which generated 25 psi of pressure. Aerosolization proceeded until the complete inoculum was expelled from the nebulizer cup (10–15 min). Sixteen of the animals inoculated with FMDV-WT were euthanized at predetermined time points for harvest of tissue samples. Time points corresponded to 0.1 (immediately after inoculation), 3, 6, 12, 24, 48, 72 or 96 h post aerosolization (hpa) (Table 1). For animal experiemnts with FMDV-Mut, practical and ethical constraints of experimentation with livestock in a biocontainment laboratory necessitated prioritization of few time points rather than replication of the entire time course performed with FMDV-WT. The most direct limitation was the stringency of the Institutional Animal Care and Use Committee (IACUC), which ensures that use of animals in experimentation always demonstrates efforts to reduce the quantity of experimental subjects to minimum levels. Based on the findings from the initial part of the study, 2 cattle inoculated with FMDV-Mut were euthanized for harvest of tissue samples at each of three time points: 24, 48 and 72 hpa (Table 2). Seven additional animals (4 infected with FMDV-WT and 3 infected with FMDV-Mut) were monitored for up to 10 days post infection for characterization of antemortem infection dynamics.

**Table 1:** FMDV-WT tissue distribution

				Pre-viremic							Viremic					
Time point (hpa)	0.1	3		6		12		24			48		72		96	
Animal ID	931	1028	1029	1001	1002	1026	1027	930	927	694	938	960	928	929	9159	962
Clinical score	0	0	0	0	0	0	0	0	0	0	2	6	8	12	4	10
*FMDV RNA copies/*μ*l in serum*	neg	neg	neg	neg	neg	neg	neg	neg	1.3	1.5	**3.38**	**3.48**	**5.88**	**5.35**	**4.60**	3.53
Tissue sample																
Oral cavity/oropharynx																
Anterior tongue	2.81	-	-	-	-	-	-	-	-	2.66	2.65	**3.38**	**5.69**	**6.39**	**3.90**	-
Lingual tonsil	2.85	-	-	-	-	2.60	-	-	**4.63**	-	-	3.64	**2.87**	**4.87**	5.26	**3.31**
Anterior hard palate	3.06	-	-	-	-	-	-	-	-	-	*NA*	*NA*	**5.30**	**4.76**	3.58	*NA*
Palatine tonsil	2.71	-	-	-	-	-	-	-	-	-	**2.83**	-	**5.20**	**5.97**	**4.57**	**3.09**
Ventral soft palate-Caudal	-	-	-	-	-	3.49	2.94	**3.99**	**4.80**	3.40	-	3.26	**4.96**	**3.86**	**4.98**	**4.12**
Nasal cavity/Nasopharynx																
Nasal turbinates-posterior	2.97	-	-	-	-	-	-	NA	+	**3.77**	2.61	2.76	**2.69**	**3.01**	**4.03**	2.65
Dorsal soft palate -Rostral	2.70	3.40	-	-	**3.91**	**2.56**	-	**4.57**	**3.13**	**3.28**	**3.57**	**4.20**	**4.08**	**3.79**	**3.96**	**3.84**
Dorsal soft palate -Caudal	3.00	4.39	-	-	3.26	**4.57**	**4.61**	**5.02**	**5.58**	**3.36**	**5.13**	**3.76**	**2.92**	**4.95**	**4.77**	**4.52**
Dorsal nasopharynx -Rostral	2.77	**3.73**	**3.97**	**4.03**	**3.64**	**3.80**	**4.07**	**5.34**	**3.11**	**4.80**	**5.47**	**6.07**	**4.26**	**4.21**	**5.39**	**4.40**
Dorsal nasopharynx -Caudal	2.78	3.61	2.94	-	**4.41**	**3.09**	**4.25**	**6.42**	**4.90**	**3.85**	**4.85**	**3.88**	**5.16**	**5.11**	**4.06**	**4.84**
Nasopharyngeal tonsil	-	-	-	-	-	-	-	**3.06**	**3.58**	3.35	3.30	**3.43**	**3.21**	**2.94**	**2.94**	-
Ventral epiglottis	3.13	2.80	-	-	-	**4.21**	3.05	**3.65**	**4.29**	-	3.05	3.07	**4.63**	**5.39**	**4.48**	**3.70**
Larynx	2.57	**3.00**	3.84	-	**4.56**	**3.05**	**4.26**	**4.18**	**3.49**	**4.20**	**3.20**	**3.97**	**3.41**	**4.00**	**4.22**	**3.31**
Lungs/Trachea																
Trachea −10 cm	2.69	-	-	3.20	-	-	2.70	-	-	-	3.13	**6.37**	**4.64**	**2.58**	4.09	**3.71**
Proximal cranial lobe	2.78	3.69	-	-	2.72	3.77	4.26	**5.18**	**3.12**	**5.35**	**6.75**	**7.15**	**4.82**	**6.09**	**5.27**	*NA*
Mid cranial lobe	3.25	3.14	-	3.87	-	2.65	**5.12**	**3.79**	**4.59**	**3.71**	**7.05**	**6.65**	-	**3.69**	**5.80**	*NA*
Distal cranial lobe	2.99	4.08	2.69	**3.78**	-	-	**5.50**	**6.10**	**2.64**	**5.91**	**7.25**	**8.18**	**3.05**	**3.48**	**7.10**	*NA*
Proximal mid lobe	2.91	3.28	-	-	-	**4.51**	**4.64**	**5.72**	**2.91**	**5.77**	**6.13**	**7.08**	**5.84**	**4.73**	4.09	**5.84**
Mid mid lobe	2.93	**3.21**	-	-	-	**3.64**	**4.43**	**5.21**	**4.10**	**5.02**	**6.56**	**6.35**	4.28	**3.78**	4.22	**6.73**
Distal mid lobe	2.99	4.52	2.86	**3.12**	3.71	**3.72**	**4.43**	**6.42**	**2.74**	**4.79**	**7.01**	**7.99**	**4.00**	**3.87**	4.21	**5.64**
Proximal caudal lobe	-	*NA* ^*a*^	*NA*	3.20	2.52	**3.73**	**4.52**	**5.67**	**3.72**	**4.91**	**6.29**	*NA*	**4.85**	**5.64**	**6.19**	*NA*
Mid caudal lobe	-	*NA*	*NA*	4.27	-	**4.33**	**4.79**	**5.70**	**3.14**	**4.16**	**6.02**	*NA*	2.55	**3.62**	5.52	*NA*
Distal caudal lobe	-	*NA*	*NA*	**2.71**	3.02	-	-	**4.90**	+	**5.47**	**5.49**	*NA*	-	**2.97**	**5.53**	*NA*
Additional tissues																
Interdigital cleft	-	-	-	-	-	-	-	-	-	*NA*	2.58	-	**6.26**	**8.97**	**5.03**	**9.22**
Medial Retropharyngeal LN	-	-	-	-	-	**+**	**+**	**2.94**	+	*NA*	**2.97**	**3.87**	**4.36**	**+**	**3.40**	+
Submandibular LN	-	-	-	-	-	-	-	-	-	*NA*	-	**4.09**	**3.24**	**3.79**	**4.17**	**4.01**
Hilar LN	-	-	-	-	**-**	**+**	**+**	**2.92**	-	*NA*	**4.80**	**5.17**	**2.81**	3.23	**3.77**	3.22

Tabulated Numbers indicate FMDV RNA genome copy numbers (GCN) per mg of tissue

Bold and underlined text indicates infectious virus isolated

‘+’ indicates infectious virus isolated without concurrent detection of FMDV genome

‘-’ indicates negative for detection of FMDV RNA and infectious virus

Additional organs collected were liver, spleen, thymus, thyroid, kidneys and heart; there was no detection of FMDV prior to onset of viremia at any of these sites

^a^NA = not assayed

**Table 2:** FMDV-Mut tissue distribution

Time point (hpi)	24		48		72	
Animal ID	699	9106	9107	1003	1024	1035
Clinical score	0	0	0	0	0	0
FMDV RNA copies/μl in serum	neg	neg	neg	neg	neg	neg
Tissue sample						
Oral cavity/oropharynx						
Anterior tongue	-	-	-	-	-	-
Lingual tonsil	-	-	-	-	3.15	-
Anterior hard palate	NA	-	-	-	-	-
Palatine tonsil	NA	-	-	-	**3.14**	-
Ventral soft palate-Caudal	-	-	-	-	**3.87**	-
Nasal cavity/Nasopharynx						
Nasal turbinates-posterior	-	-	-	-	-	-
Dorsal soft palate -Rostral	+	2.85	3.16	-	3.73	-
Dorsal soft palate -Caudal	**2.68**	**4.74**	**4.15**	**2.83**	**4.38**	**2.95**
Dorsal nasopharynx -Rostral	**4.43**	-	-	-	**3.01**	**3.07**
Dorsal nasopharynx -Caudal	+	2.57	**4.54**	-	**3.42**	3.05
Nasopharyngeal tonsil	-	-	-	+	-	-
Ventral epiglottis	-	-	**4.31**	-	4.10	-
Larynx	2.70	-	2.75	**5.12**	**3.10**	-
Lungs/Trachea						
Trachea −10 cm	-	-	-	-		-
Proximal cranial lobe	NA	-	**3.47**	4.09	**4.79**	2.96
Mid cranial lobe	NA	-	**3.86**	3.13	2.68	3.91
Distal cranial lobe	**3.36**	2.57	+	3.00	**4.26**	3.98
Proximal mid lobe	NA	-	**3.95**	3.22	**5.55**	3.90
Mid mid lobe	NA	-	**5.56**	3.52	4.25	3.92
Distal mid lobe	**3.78**	-	3.47	-	3.54	**3.22**
Proximal caudal lobe	NA	-	**4.99**	4.08	**4.75**	3.73
Mid caudal lobe	NA	3.49	-	3.40	**4.27**	-
Distal caudal lobe	**3.62**	3.04	**3.43**	-	3.31	-
Additional tissues						
Interdigital cleft	NA	-	-	-	-	-
Medial Retropharyngeal LN	NA	-	-	-	+	-
Submandibular LN	NA	-	-	-	**2.57**	-
Hilar LN	NA	-	-	-	**2.98**	-

Tabulated Numbers indicate FMDV RNA genome copy numbers (GCN) per mg of tissue

Bold and underlined text indicates infectious virus isolated

‘+’ indicates infectious virus isolated without concurrent detection of FMDV genome

‘-’ indicates negative for detection of FMDV RNA and infectious virus

### Sample collection

Clinical (antemortem) sampling consisted of collection of whole blood in serum separation tubes, and oral and nasal fluids with cotton swabs. Animals were sampled at several time points which varied according to goals of the individual experiments. Swabs and serum tubes were transported from the animal rooms to the laboratory on ice and were immediately centrifuged for harvesting of serum, saliva, and nasal secretion. Samples were subsequently stored at −70 °C until time of processing. Clinical scores were based on a 20 point scale accounting for presence of vesicles on each foot and anywhere on the head (oral cavity or nasal epithelia) as previously described [9, 12].

Cattle were euthanized at predetermined time points regardless of clinical progression of disease. Postmortem sample collection schemes were standardized with variation among individual animals based upon the expected stage of disease at the time of euthanasia. Detailed descriptions of tissue designations and collection strategies has been published previously [9, 12]. Tissues analyzed included predefined segments of nasopharynx (distinct rostral and caudal specimens from dorsal soft palate and roof of nasopharynx), lungs (9 distinct specimens per animal from different lung lobes and distinct segments of each lobe), lesion-predilection sites (tongue and foot epithelium), lymph nodes, non-lesion epithelia, and visceral organs (Tables 1 and 2).

For each tissue specimen, three 30–50 mg tissue sub-samples were aliquoted into separate 1.5 ml screw-cap tubes and frozen immediately in liquid nitrogen for transfer to a −70 °C freezer in which they were stored until the time of processing. Additional, adjacent specimens of each tissue were placed in cryomolds, embedded in Optimal Cutting Temperature Compound (OCT; Sakura Finetek, Torrance, CA), frozen over liquid nitrogen, and stored at −70 °C for immunomicroscopy.

### Foot-and-mouth disease viral RNA detection

Two samples of each tissue specimen were thawed and macerated in a TissueLyser bead beater (Qiagen, Valencia, CA) as previously described [12]. RNA was extracted with the MagMax-96 Viral RNA Isolation Kit (Ambion, Austin, TX) on a King Fisher-96 Magnetic Particle Processor (Thermo Scientific, Waltham, MA). After extraction, viral RNA (vRNA) was detected by quantitative, real-time reverse transcription polymerase chain reaction (qRT-PCR) on the ABI 7000 system (Applied Biosystems, Austin, TX) as previously described [23]. The assay used detects a segment of the highly conserved FMDV 3D protein sequence using forward primer (5′ACT GGG TTT TAC AAA CCT GTG A), reverse primer (5′-GCG AGT CCT GCC ACG GA), and probe (5′-TCC TTT GCA CGC CGT GGG AC). Samples with cycle threshold (Ct) values < 40 were considered positive. Clinical samples (serum and swabs) were processed similarly with the exception that a single extraction was performed on each sample and subsequently used for 2 replicate qRT-PCR reactions. Ct values generated by FMDV qRT-PCR from experimental specimens were converted to FMDV RNA genome copy numbers (GCN) per mg or μl as previously described [2, 9]. The Ct positivity cutoff corresponded to a detection threshold value of 2.52 log_10_ FMDV GCN/mg (RNA/mg) of tissue. FMDV qRT-PCR Ct values were expressed as the mean log_10_ FMDV RNA copies/mg (RNA/mg). For clinical samples (sera/swabs), reported data represents the geometric mean log_10_ FMDV GCN/μl from animals infected with the same virus and sampled at each time point.

### Foot-and-mouth disease virus isolation

Virus isolation (VI) was performed separately on the duplicate samples of each tissue on BHK-21 cells as previously described [24]. Subsequent to evaluation of cytopathic effect (CPE), FMDV-positivity/negativity was confirmed by qRT-PCR of cell culture supernatants. Samples which had no CPE, but from which vRNA was detected by qRT-PCR were passed a second time in BHK-21 cells.

### Immunomicroscopy

Microscopic localization of FMDV antigens and host proteins in cryosections was performed by immunohistochemistry (IHC) and multichannel immunofluorescence (MIF) microscopy as previously described [9, 13, 25]. Slides were examined with a wide-field, epifluorescent microscope, and images were captured with a cooled, monochromatic digital camera. Images of individual detection channels were adjusted for contrast and brightness and merged in commercially available software (Adobe Photoshop, CS2). Mouse monoclonal anti-FMDV-VP1 has been described previously [26]. Antibodies used to label cell markers in MIF experiments were mouse monoclonal anti-pancytokeratin plus (Biocare CM162), anti-MHCII (AbD Serotec, MCA2225PE), and anti-CD11c (VMRD No. BAQ153A, VMRD, Pullman, WA).

For every IHC and MIF experiment, a duplicate, negative-control serial section treated with an isotype-matched irrelevant antibody or isotype control reagent of similar concentration was included. MIF labeling was considered positive when there was an intense cell-associated signal within the experimental tissue with the absence of such staining in the negative controls.

### Ethics of animal use

Experiments involving live animals were performed under protocol 209-02-07-R approved by the Institutional Animal Care and Use Committee of the Plum Island Animal Disease Center. This experimental protocol delineates humane endpoints at which animals are to be euthanized to abrogate suffering. These criteria were not met by any animals included in this study and there were no unexpected animal deaths. All animals were euthanized at pre-determined time points by intravenous injection of an overdose of pentobarbital (90 mg/kg) following sedation by intramuscular injection of Xylazine hydrochloride (0.66 mg/kg). Clinical examinations and inoculations were performed after sedation by Xylazine as described above with reversal by intravenous injection of Tolazoline (2.0 mg/kg). Cattle that developed marked lameness due to clinical FMD were treated by intramuscular administration of flunixin meglumine (Banamine^©^; 2.2 mg/kg) at 24 h intervals.

## Results

In order to characterize the pathogenesis of FMD in cattle, a series of experiments was performed in which steers were exposed to FMDV under simulated-natural conditions. The scheme of all experiments was similar including periodic antemortem sampling (sera and swabs), clinical scoring at 24 h intervals, and euthanasia with postmortem tissue collections. An intensive time-course was conducted using a wild-type FMDV infectious clone (FMDV-WT) with a more limited set of experiments performed with the attenuated mutant virus (FMDV-Mut) focusing on the critical pathogenesis landmarks. Specifically, time points for the experiments with FMDV-Mut were chosen based upon three distinct phases identified after infection with FMDV-WT: 24hpi primary infection, 48 h early viremia, 72 h fulminant viremia and clinical disease. In all experiments, the presence of infectious FMDV in tissues and fluids was determined by virus isolation, whereas viral loads were determined and compared using quantitative real time PCR (qRT-PCR).

### FMDV-WT; Clinical signs and antemortem viral dynamics

**Fig. 1:**
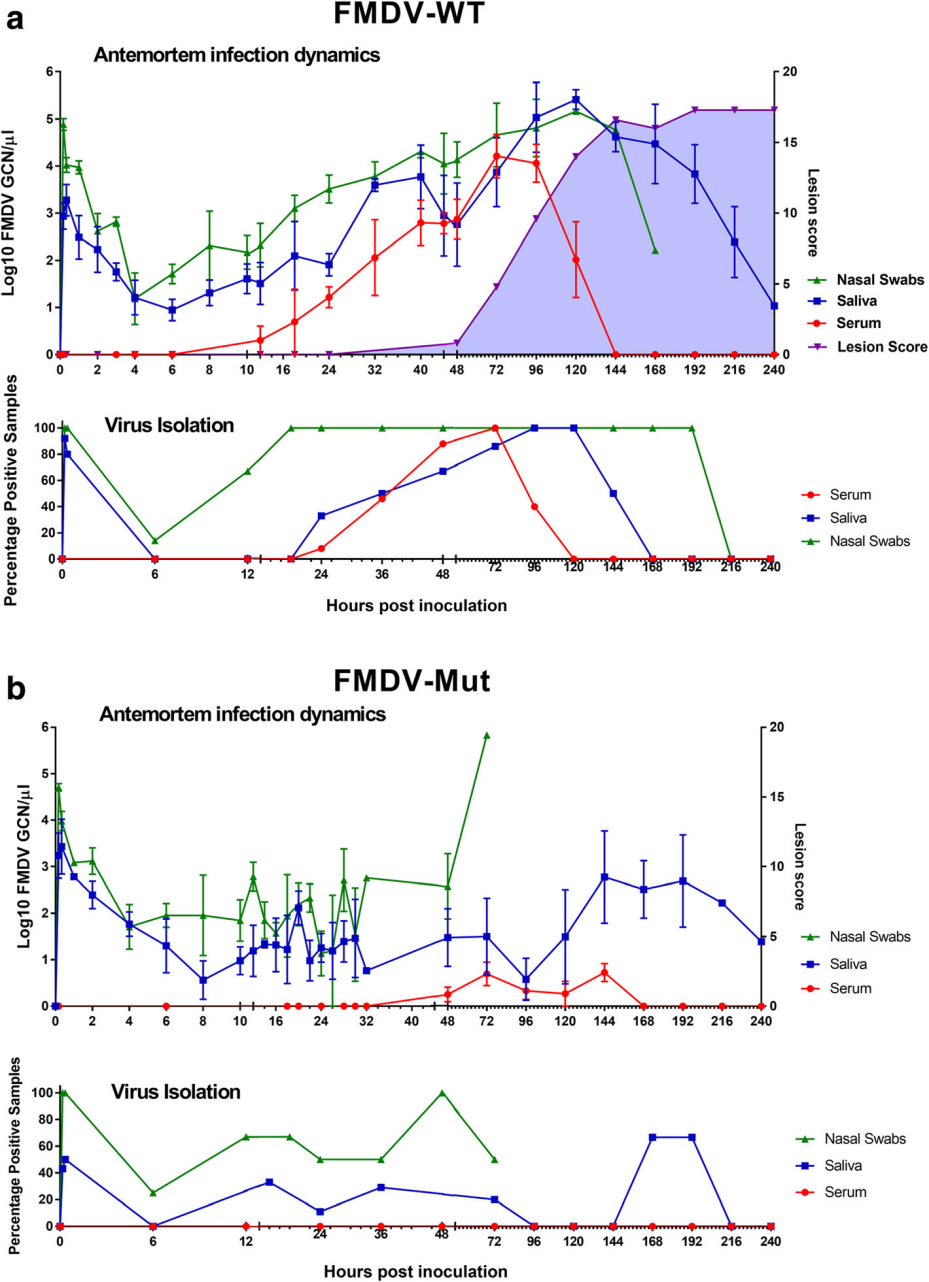
Antemortem infection dynamics in steers following aerosol-inoculation with distinct strains of FMDV-A24-Cruzeiro: **a ** FMDV-WT (*n* = 20) or **b ** FMDV-Mut (*n* = 9). **a** & **b** FMDV RNA detection in serum, oral and nasal swabs was performed through qRT-PCR, and is presented as log_10_ genome copy numbers (GCN)/ul. Data presented are average values (mean +/− SEM) based on samples collected from all cattle included at each time point. Virus isolation (VI) was performed on BHK-21 cells and is presented as the percentage of VI positive samples per time point. High GCN/ul of RNA and VI percentage positivity immediately after inoculation indicates detection of residual inoculated virus. * X-axes scales are non-linear. For FMDV-Mut, nasal swab data is derived entirely from animals 699, 9106, 9107, 1003, 1024, and 1025

Immediately after aerosol-inoculation, high quantities of vRNA, corresponding to residual inoculum, were detected in nasal and oral secretions of all animals (Fig. 1a). This detection gradually decreased for 4 h (nasal) or 6 h (oral) until vRNA loads began to increase suggesting detection of the *de novo* replication of FMDV (Fig. 1a). Thus, the mean onset of detection of FMDV replication in secretions was 5hpa. Once *de novo* replication was detected, vRNA levels in secretions rose continuously throughout and beyond the incubation period (0-48hpa), reaching maximum levels at 120hpa with mean detection levels of 5.17 log10 RNA/μl in nasal secretions and 5.41 log10 RNA/μl in saliva. Infectious FMDV was isolated from most nasal and saliva samples collected after 10hpa with greater prevalence of detection in nasal as compared to saliva samples (Fig. 1a). In serum, the overall trend was gradually increasing detection of vRNA from 12-72hpa followed by declining detection from 72-96hpa and return to negative at 144hpa. The earliest detection of vRNA in the serum of an individual animal occurred at 12hpa; however, the earliest detection of infectious virus was at 20hpa in one animal (Fig. 1a).

Clinical signs of FMD consisting of one or more vesicle(s) were first detected at 48 hpa. Fever was detected intermittently in most animals (not shown), but was a less consistent indicator of clinical phase of disease. All animals that survived to the clinical phase of FMD developed typical vesiculobullae of tongue, dental pad, interdigital epithelium, and nasal planum. Coronary band lesions were not apparent. Lesion scores progressed from 48–192 h despite cessation of viremia at 144hpa.

### FMDV-Mut: clinical signs and antemortem viral dynamics

No animals that were aerosol-inoculated with FMDV-Mut developed any clinical signs of FMD at any time (Fig. 1b). Additionally, FMDV-Mut infectious virus was never isolated from sera of infected animals; however small quantities of vRNA were detected in sera of several animals at various time points (Fig. 1b). Detection of FMDV-Mut in oral and nasal secretions was remarkably similar to detection of FMDV-WT from 0 to 12hpa (Fig. 1b). Specifically, detection of high levels of inoculated vRNA in secretions diminished from 0.1 to 6hpa followed by increasing vRNA concentrations suggesting *de novo* generated vRNA, first detected in nasal swabs at 8 hpa. However, from 18hpa till the end of the acute infection period, the magnitudes of vRNA detection diverged substantially, with consistently lower detection of the mutant virus. Despite the lower quantity of vRNA detected, infectious virus was isolated from most of the nasal swabs that contained vRNA.

### FMDV-WT: tissue-specific distribution of FMDV and viral RNA in aerosol-inoculated steers

In order to evaluate the distribution of inoculum following aerosol inoculation, one animal was euthanized 10 min after completion of aerosolization (time point 0.1 in Table 1). vRNA was detected within the oro- and nasopharynx, larynx as well as cranial and mid pulmonary lobes. However, there was no isolation of FMDV from any tissues at this early time point, demonstrating that the tissue specific loads of inoculum were below the threshold of detection of virus isolation. The early, previremic period 3–12 hpa was characterized by progressively increasing detection of vRNA and infectious virus limited to nasopharyngeal mucosal tissues and lungs (Table 1). At 3hpa, infectious FMDV and vRNA were recovered from the rostral dorsal nasopharynx of both sampled animals suggesting this tissue as the most common site of primary infection. Over successive time points, the tissue level prevalence and the viral loads increased in both upper and lower respiratory tract compartments. However, the most consistent site of detection of FMDV in the previremic period was the rostral segment of the dorsal nasopharynx which was the only tissue that was positive by qRT-PCR and VI in every previremic animal.

Coincident with the cusp of onset of viremia, the 24 hpa time point had distinct pattern of viral distribution. Although no animals euthanized at this point had infectious virus in serum, vRNA was detected within sera of 2 of the 3 animals. Tissue distribution was more extensive with 100% tissue-level prevalence in nasopharynx and lungs. At this point, FMDV was also detected in the larynx, oropharynx and lymph nodes draining the respiratory tract indicating greater regional dissemination compared to the earlier previremic time points.

Both animals euthanized at 48 hpa were viremic (defined by infectious virus in serum), but with relatively low levels of vRNA in serum. Viral loads were substantially higher in lungs compared to earlier timepoints, but were relatively stable in the nasopharynx compared to previremic levels. Additionally, there was higher prevalence of detection in lymph nodes, palatine tonsils, and distant epithelial sites. Peak viremia at 72hpa (5.9 log_10_ FMDV GCN/μl) was associated with the broadest distribution of detection of FMDV in tissues. Because of the extensive intravascular FMDV in tissues during viremia it was not possible, based upon qRT-PCR and VI, to definitively discern the relative contributions of intravascular virus versus regional replication. Thus, microscopic localization of FMDV antigens (described below) was critical to ascertain pathogenesis events at this time point. However, it is noteworthy that despite the increased quantity of FMDV within vessels and many tissues, the viral loads in pulmonary tissues were substantially lower than at 48 hpa. By 96hpa, viremia and detection of FMDV in tissues was waning. The decreased viral load in serum was associated with a lower prevalence of FMDV-containing tissues. However, many of the tissues that remained positive had extremely high viral loads, particularly in the nasopharynx and lungs.

### FMDV-Mut: tissue-specific distribution of FMDV and viral RNA in aerosol-inoculated steers

A more limited series of tissue collection experiments using FMDV-Mut was focused on the critical virulence-defining events in pathogenesis of FMDV between 24 and 72 hpa. Specifically, these experiments addressed the hypothesis that the continuum between attenuation and virulence of FMDV could be elucidated by characterizing viral dynamics in tissues and secretions subsequent to infection with the two viruses.

The distribution and viral loads associated with infection with FMDV-Mut was remarkably similar across the three time points examined (Table 2). The most consistent detection occurred in the nasopharynx at the caudal segment of the dorsal soft palate which was the only tissue that was positive for vRNA and infectious virus in every animal examined. At each timepoint there was one animal that had substantially greater detection in pulmonary tissues demonstrating permissiveness, but variability of infection of pulmonary tissues. Tissue-level prevalence of detection increased over the time course, but viral loads remained relatively unchanged. Unlike FMDV-WT there was neither viremia nor dissemination of FMDV-Mut to distant tissues. The only detection of FMDV-Mut beyond mucosal surfaces and lungs occurred in one animal at 72hpa in palatine tonsil and submandibular and retropharyngeal lymph nodes. Mean viral loads of FMDV-Mut in nasopharynx and lungs were lower than those detected for FMDV-WT at every corresponding time point [2].

### FMDV-WT: microscopic characterization of tissues of aerosol-inoculated cattle

**Fig. 2:**
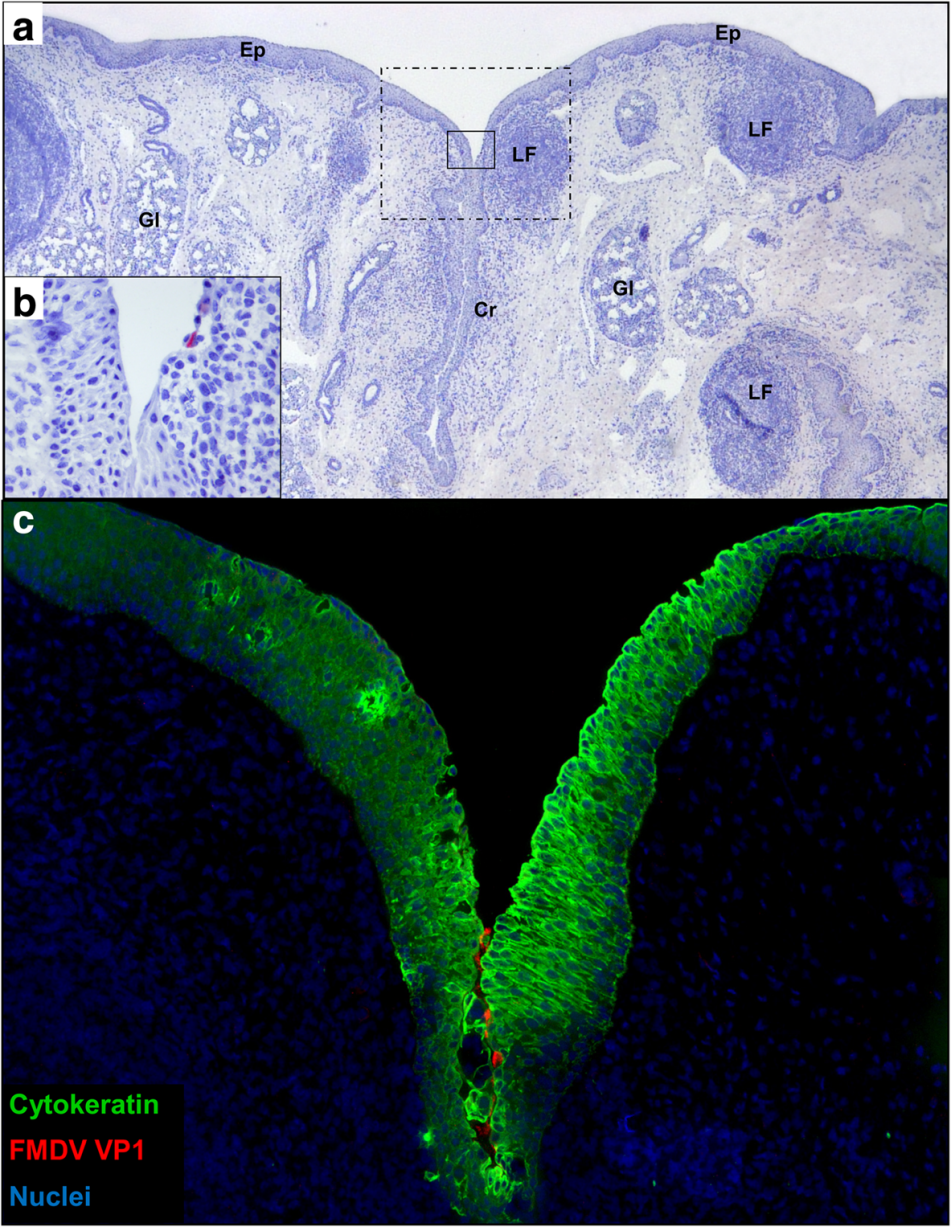
Detection of FMDV-WT by immunomicroscopy in bovine nasopharynx at 6 h post aerosol-inoculation. **a** Low magnification view demonstrates tissue architecture of the caudal aspect of the dorsal soft palate including surface epithelium (Ep), epithelial crypts (Cr), lymphoid follicles (LF) of mucosa-associated lymphoid tissue, and secretory glands (Gl). Two regions of interest (ROI) are centered on an epithelial crypt with a prominent subepithelial lymphoid follicle: *solid-lined box* indicates ROI highlighted in 1**b**; *dashed box* indicates ROI highlighted in 1**c**. Immunohistochemistry (IHC) with anti-FMDV capsid monoclonal antibody, micropolymer alkaline phosphatase detection system with hematoxylin counterstain. **b** Earliest microscopic detection of FMDV consisting of few cells containing FMDV VP1 within a shallow depression within the wall of an epithelial crypt. IHC with anti-FMDV capsid monoclonal antibody, micropolymer alkaline phosphatase detection system. **c** Serial section of tissue shown in **a**-**b** demonstrating few cells containing FMDV VP1 (*red*) within epithelium (*green*) or adhered to epithelial surface of crypt. Multichannel immunofluorescence microcopy, (animal ID 1002, tissue ID Dorsal soft palate -Caudal) (magnification: **a** 2×, **b** 40×, **c** 10×)

The earliest microscopic localization of FMDV-WT occurred within nasopharyngeal mucosa at 6hpa (Fig. 2). At this time point, few FMDV-immunopositive cells were identified within the superficial epithelium at the opening of an epithelial crypt overlying a lymphoid follicle (mucosa associated lymphoid tissue; MALT). The region was rarefied and slightly concave suggesting cell-loss (erosion). Examination of the region with simultaneous multichannel immunofluorescence (Fig. 2c) indicated that the cells containing FMDV antigens were predominantly cytokeratin + consistent with epithelial histogenesis. FMDV could not be microscopically localized to any other tissue at this time point.

**Fig. 3:**
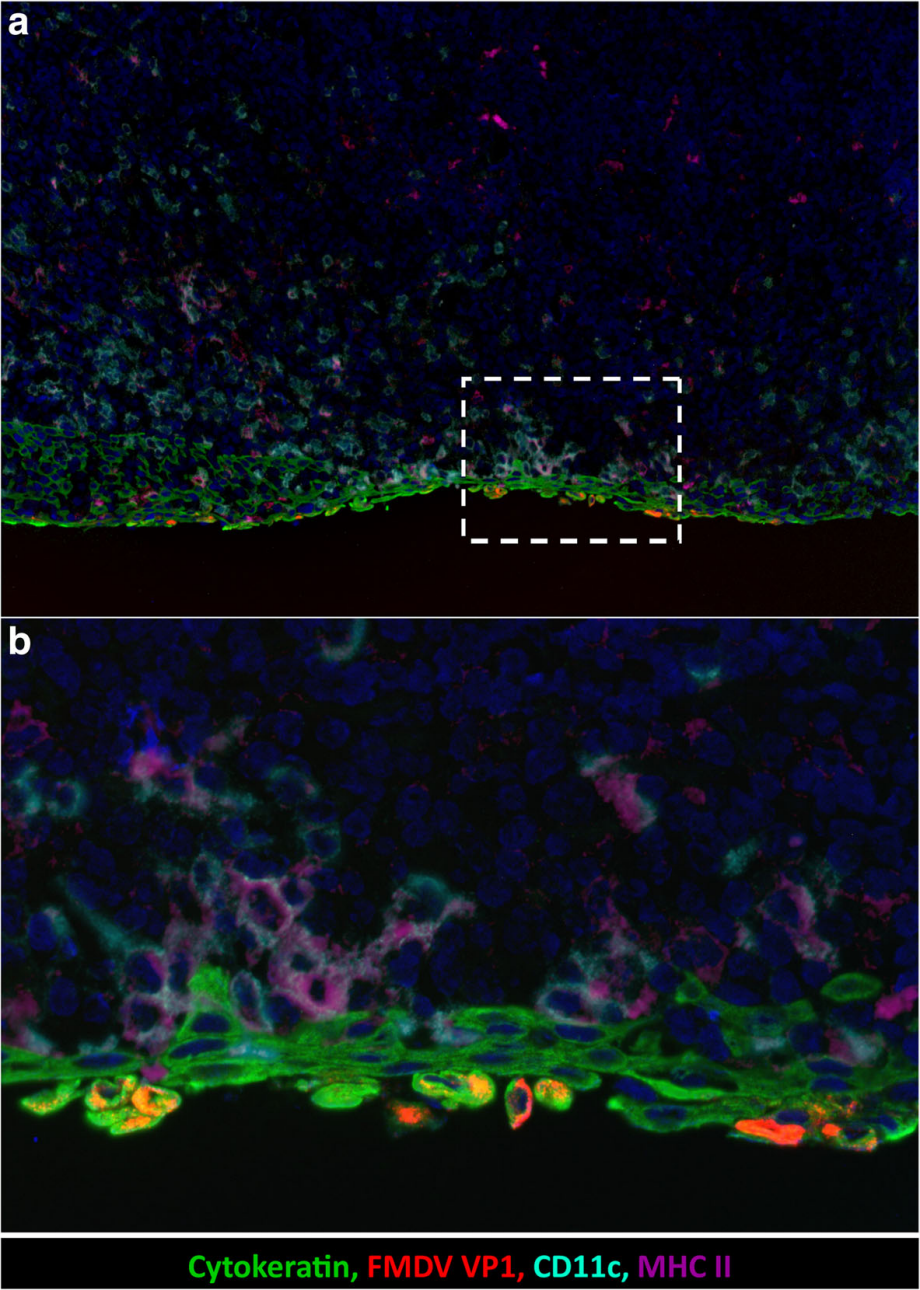
Detection of FMDV-WT by immunomicroscopy in bovine nasopharynx at 12 h post aerosol-inoculation. **a** Cells containing FMDV VP1 antigen (*red*) are within the cytokeratin-positive epithelium (*green*). Epithelium overlies an expansive field of mucosa-associated lymphoid tissue with numerous cells containing CD11c (turquoise) and MHCII (*purple*). *Dashed-lined box* indicates region of interest (ROI) shown at higher magnification in 3**b**. **b** FMDV-containing cells express cytokeratin and are in the superficial-most layer of epithelium. These cells have morphology consistent with acantholytic degeneration including swelling and dissociation from adjacent cells. Multichannel immunofluorescence microcopy, (animal ID 1027, tissue ID Dorsal nasopharynx -Rostral) (magnification: **a** 10×, **b** 40×)

**Fig. 4:**
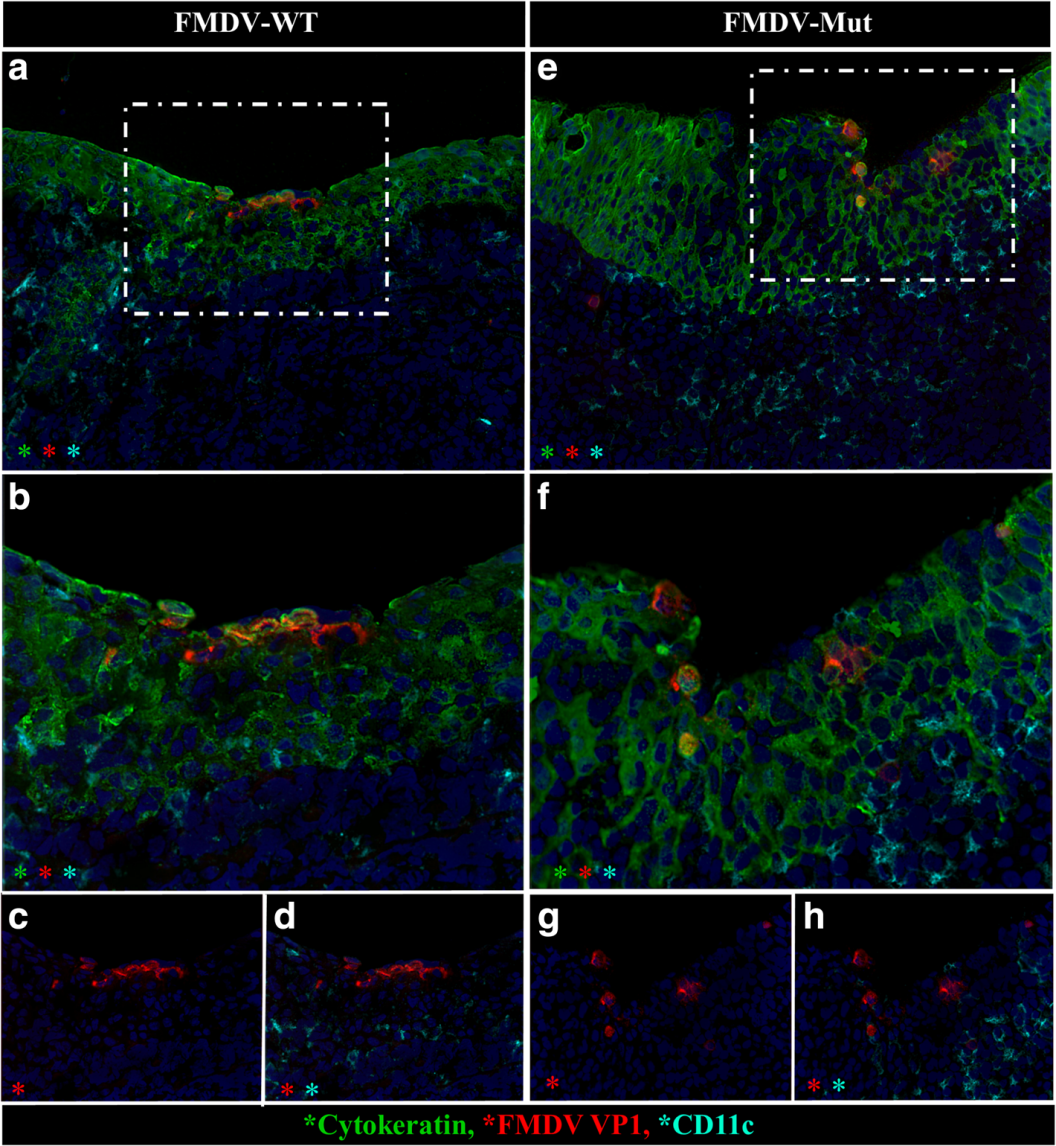
At 24 hpa, distribution and morphology of primary infection is similar for FMDV-WT and FMDV-Mut. Microscopic distribution of FMDV structural protein VP1 (*red*) cytokeratin (*green*), and CD11c (turquoise) in nasopharyngeal mucosa of cattle infected with FMDV-WT (**a**-**d**) or FMDV-Mut (**e**-**h**). **a** & **e** Both viruses similarly infected superficial cytokeratin-positive nasopharyngeal mucosal epithelial cells. *Dashed-lined boxes* indicate regions of interest (ROI) shown at higher magnification in **b**-**d**, **f**-**h**. **b**-**d** and **f**-**h** Higher magnification and selective channel combinations demonstrate similar co-localization patterns for both viruses. FMDV VP1 (*red*) predominantly localizes within cytokeratin-positive epithelial cells (*green*), but not with CD11c (turquoise). Few infected cells contain neither cytokeratin nor CD11c (phenotype undetermined). Multichannel immunofluorescence microcopy, (FMDV-WT animal ID 930, tissue ID Dorsal nasopharynx –Rostral; FMDV-Mut animal ID 699, tissue ID Dorsal soft palate -Caudal) (magnification: **a** & **e** 20×, **b**-**d** & **f**-**h** 40×)

At 12-24hpa FMDV was similarly localized to multifocal segments of nasopharyngeal epithelial MALT regions (Figs. 3 and 4). Similar to 6hpa, FMDV-immunoreactive cells were uniformly cytokeratin-positive epithelial cells. However, unlike 6hpa, there were more abundant FMDV-infected cells and they localized exclusively to the flat, expansive epithelial dome regions overlying large, cryptless lymphoid follicles. From 24 to 48 hpi, erosive indentations were occasionally present overlying nasopharyngeal MALT follicles. And, from 48 to 72hpi, FMDV VP1 was localized within CD11c + cells in MALT lymphoid follicles (not shown).

**Fig. 5:**
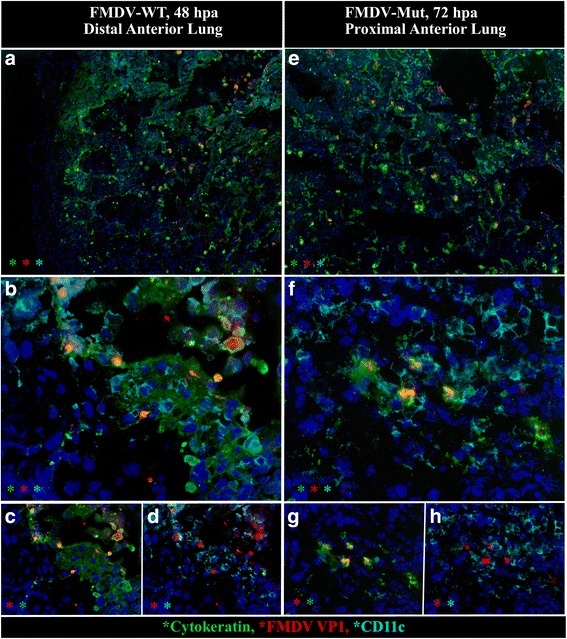
Distribution and morphology of pulmonary infection was similar for FMDV-WT at 48hpa and FMDV-Mut at 72hpa. Microscopic distribution of FMDV structural protein VP1 (*red*), cytokeratin (*green*), and CD11c (turquoise) in lungs of cattle infected with FMDV-WT (**a**-**d**) or FMDV-Mut (**e**-**h**). **a** & **e** Between 48 and 72hpa both viruses generated poorly demarcated foci of infected cells, and similarly formed vesicle-like cavitations comprised of acantholytic cells and debris. Foci of infection were more abundant in animals infected with FMDV-WT. **b**-**d** and **f**-**h** Higher magnification and selective channel combinations demonstrate similar co-localization patterns for both viruses. FMDV VP1 (*red*) predominantly localizes within cytokeratin-positive pulmonary epithelial cells (*green*), but not with CD11c monocytoid cells (turquoise). Acantholytic cells are cytokeratin-positive. Few infected cells contain neither cytokeratin nor CD11c (phenotype undetermined). Multichannel immunofluorescence microcopy. (FMDV-WT animal ID 960, tissue ID Lung; FMDV-Mut animal ID 1027, tissue ID Lung) (magnification: **a** & **e** 10×, **b**-**d** & **f**-**h** 40×)

FMDV-WT was localized to pulmonary epithelial cells starting at 12hpi (not shown). At this timepoint, viral antigens were localized to intact cells lining the alveolar septa. Over subsequent timepoints, FMDV+ alveolar septal cells continued to be present, however there were increasing quantities of FMDV+ free cells within alveolar spaces (Fig. 5a-d). These cells were consistently cytokeratin+/MHC-/CD11c- suggesting that they were degenerate (acantholytic) pnuemocytes rather than antigen presenting cells or macrophages. Pulmonary lesions progressed throughout the viremic period often forming regionally extensive, vesicle-like regions in which alveolar septae were denuded with abundant pnuemocytes undergoing acantholytic degeneration (Fig. 5).

**Fig. 6:**
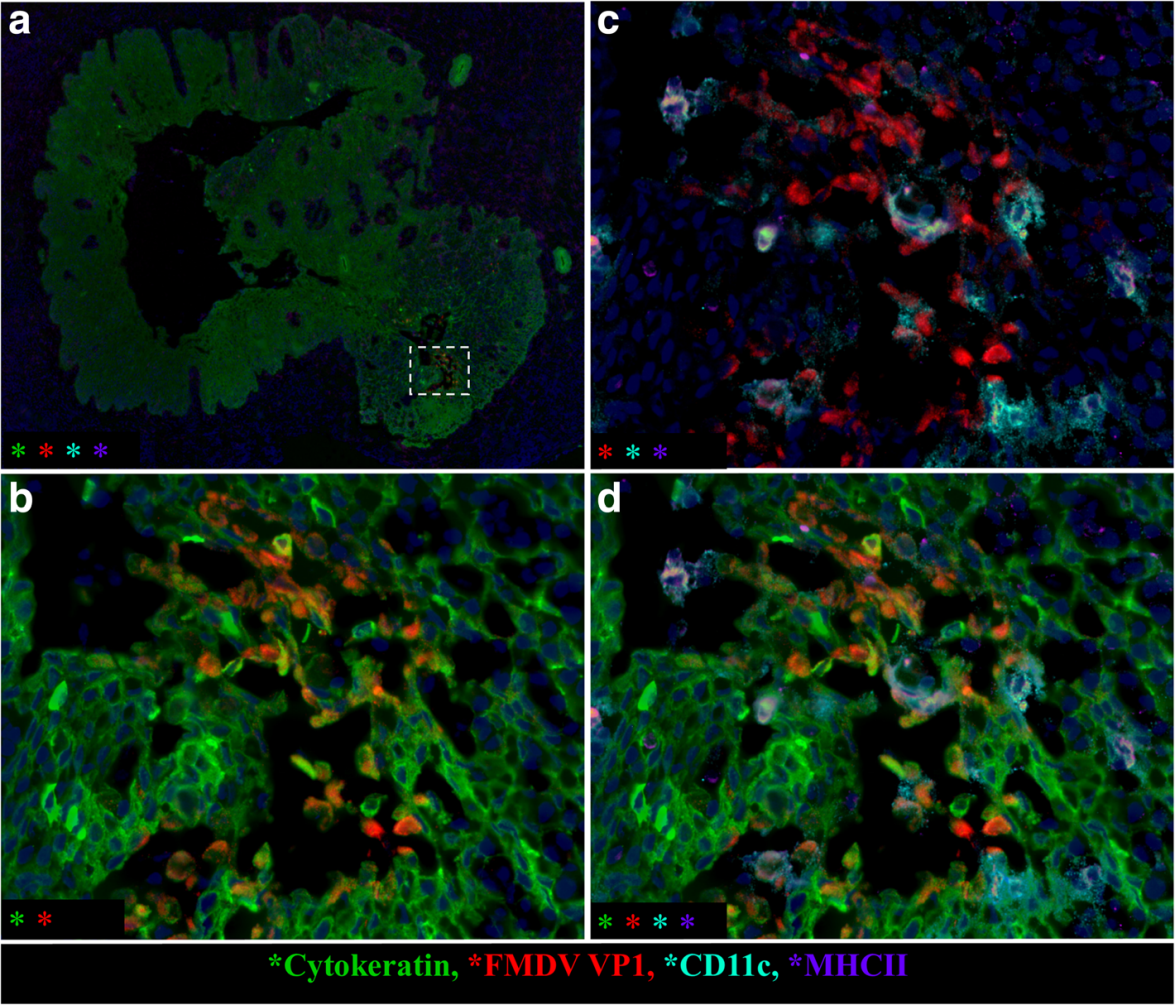
FMDV generates microvesicles within the palatine tonsil of cattle during the clinical phase of disease. **a** Low magnification image demonstrates the architecture of a large tonsillar crypt delineated by cytokeratin-positive (*green*) squamous epithelium. *Dashed box* indicates region of interest (ROI) within crypt wall containing a focus of FMDV (*red*)-infected cells forming a microvesicle. ROI is featured at higher magnification in 6**b**-**d** including different detection channels in each panel. **b** Inclusion of FMDV VP1 (*red*) and cytokeratin (*green*) channels demonstrates disruption of epithelial architecture with cavitation/vesiculation. Many of the FMDV-containing cells are also cytokeratin-positive (epithelial cells). **c** Inclusion of FMDV VP1 (red), CD11c (turquoise), and MHC II (*purple*) demonstrates that some of the FMDV-containing cells are also individually- or double-positive for these markers of monocytoid, phagocytic, and antigen presenting cells. **d** Simultaneous viewing of all 4 channels demonstrates that within the vesicular cavity, FMDV-containing cells of distinct phenotypes are interspersed and in close proximity. Multichannel immunofluorescence microcopy. (animal ID 938, tissue ID palatine tonsil) (magnification: **a** 4×, **b**-**d** 40×)

The palatine tonsil was the only non-respiratory tissue in which FMDV antigens were localized by immunomicroscopy. At 48-72hpi, there were regionally extensive intra-epithelial vesicles within the stratified squamous epithelium of palatine tonsils that had all the conventional attributes of classical FMD vesicles, including acantholytic degeneration of epithelial cells, cavitation, and limitation of infection to cytokeratin-positive cells (Fig. 6).

### FMDV-Mut: microscopic characterization of tissues of aerosol-inoculated cattle

The morphologic and phenotypic characteristics of infection of the bovine respiratory tract with FMDV-Mut between 24 and 72 hpa were essentially identical to the description of infection with FMDV-WT. However, at all comparable time points, substantially fewer foci of infection could be identified in tissues of animals infected with the mutant virus compared to FMDV-WT. Similar to FMDV-WT, the earliest localization of FMDV-Mut was detected in the cytokeratin-positive, epithelial cells overlying lymphoid follicles of nasophayngeal MALT (Fig. 4d-f). This was detected at 24hpi which was the earliest tissue sampling point. At 72hpi FMDV structural antigen (VP1) was detected within lymphoid follicles and surface epithelium of nasopharyngeal MALT tissue (not shown). Additionally vesicle-like regions of pulmonary acantholytic degeneration were detected at 48–72 hpi in cattle inoculated with FMDV-Mut (Fig. 5e-h). Within these regions, FMDV colocalized with cytokeratin-containing cells within alveolar lumens and septa.

## Discussion

The functional genomics of the FMDV leader proteinase (L_pro_) have been investigated in various manners since the landmark studies by Brown et al. which defined L_pro_ as the first identified FMDV virulence factor in cattle [1]. Subsequent works have further defined the importance of L_pro_ by demonstrating complete or partial attenuation of leaderless FMDVs in pigs [18, 27]. Additionally, insertion mutagenesis studies have demonstrated that certain in-frame disruptions of L_pro_ were sufficient to generate a virus with attenuated phenotype in cattle [3].

Various studies have demonstrated functional attributes of FMDV L_pro_ using in vitro systems [28, 29]. Subsequent work by Belsham delivered a precise map of attenuating and lethal mutations within the L_pro_ coding region through a combination of deletions and site-directed mutagenesis [4]. However, mechanisms of attenuation suggested by in vitro work have not been substantiated in vivo. Functional investigations performed in natural host species within our laboratory, indicated that cattle that were aerosol-inoculated with leader-attenuated FMDV were induced to transcribe interferon mRNA, but failed to mount an effective antiviral response [2]. That previous publication provided transcriptomic details of the host response and summary- level comparisons of viral dynamics in a limited set of short-duration experiments; however tissue-specific viral quantitation and microscopic localization was not examined. In the current work, the continuum of virulence and attenuation of leader-mutagenized FMDV was investigated in cattle through an extensive series of experiments in which cattle were inoculated by a simulated natural route (aerosol) with either a mutagenized FMDV or the parental infectious clone (FMDV-WT) from which the mutant virus was derived. The ultimate output was that despite the attenuated phenotype, FMDV-MUT was able to achieve some of the early pathogenesis landmarks of virulent FMDVs.

In order to validate the functional deficiencies of the leader-mutagenized virus (FMDV-Mut), it was necessary to first demonstrate that the parental infectious clone virus (FMDV-WT) was functionally similar to the field strains from which it was derived. This was accomplished through a series of aerosol-inoculation experiments which were terminated by euthanasia and tissue harvests at 0.1-96 hpa. These experiments demonstrated that the infectious clone virus generated a fulminant FMD phenotype and viral dynamics which were highly consistent and similar to that observed subsequent to aerosol-inoculation of FMDV-O1-Manisa [9]. Additionally, the FMD syndrome that resulted from aerosol inoculation of FMDV-WT was similar to that produced by an in vivo-derived (i.e., not cloned), field variant of FMDV-A24-Cruzeiro inoculated by nasopharyngeal deposition [13] or direct contact exposure [30]. Overall, the findings of the current work support the previously established landmarks of bovine FMD pathogenesis as 1) Primary infection within the nasopharyngeal mucosa, 2) Variable pulmonary phase which is more prominent in aerosolized animals, 3) Viremia and clinical FMD including fever and vesiculation.

The clinical phenotype of FMDV-Mut was consistently completely attenuated despite numerous subclinical similarities to FMDV-WT. None of the animals inoculated with FMDV-Mut had fever or vesicles at any time. For both viruses, the period of 0-6hpi was characterized by clearance of the inoculum from the upper respiratory tract, presumably through the mucociliary apparatus. The detection nadir of viral RNA in nasal secretions of animals inoculated with FMDV-Mut was 1.9 log_10_ less than FMDV-WT suggesting that within hours of infection the mutant virus was already demonstrably impeded from replicating effectively. Overall, the temporal patterns and locations of detection of FMDV-Mut in live animals were suggestive of a virus that had retained most of the functionality of typical FMDVs, but with blunted efficacy (virulence). This is consistent with characteristics of FMDV-Mut described in vitro which included preservation of most functionalities of the parental virus including auto-catalysis of L_pro_ and ability to cleave the host translation initiation factor eIF4G [3]. Additionally, viral protein expression profiles in vitro were similar between the two viruses, however expression was delayed in the mutant relative to the parental virus. FMDV-Mut grew more slowly in tissue culture, and had a smaller plaque size phenotype than the parental virus [3].

The tissue-specific detection of the two FMDVs in the current study and their viral RNA further delineated the similarities and differences between the viruses. FMDV-WT demonstrated consistent tropism for nasopharyngeal mucosal tissues in every animal examined including those euthanized at 3hpi. To our knowledge this is the earliest reported recovery of FMDV from tissues of an infected animal. The specific anatomic sites with the greatest prevalence and quantities of previremic detection of FMDV were the rostral segment of the dorsal nasopharynx and caudal segment of the dorsal soft palate. Limited respiratory tract distribution at 3-6hpi led to pan-respiratory generalization from 12-24hpi which was followed by systemic generalization which progressed from 48-96hpi. During the systemic phase of disease, FMDV and vRNA were recovered from almost every tissue examined due to detection of virus within the vasculature.

Tissues collected from FMDV-Mut infected cattle at 24-72hpi had substantial similarity to tissues from FMDV-WT infected animals at 3-12hpi (previremic phase). The consistent infection of the nasopharyngeal sites, relatively low viral loads, and inconsistent respiratory generalization including variable pulmonary involvement were all consistent with the earliest time points of infection with FMDV-WT. Similar to FMDV-WT, oropharyngeal sites (ventral soft palate, palatine tonsil, and hard palate) did not support viral replication. Nasopharyngeal tropism has previously been demonstrated for attenuated FMDVs with deletions in 3A gene [6]. However, unlike FMDV-WT’s anatomic and quantitative progression up to 48hpi, FMDV-Mut distribution and viral loads remained relatively static across the study period. Several significant quantitative differences were identified between the tissue-specific viral loads during 24-72hpi period [2].

In the current study, microscopic immunolocalization in bovine tissues further confirmed the similar distribution and tropism of the virulent and attenuated FMDVs. The earliest microscopic detection of FMDV-WT antigen was at 6hpi in the epithelial cells of specialized regions of epithelium overlying nasopharyngeal MALT. The tropism for epithelium was confirmed by co-localization of FMDV antigens with cytokeratin. This follicle-associated epithelium (FAE) has previously been demonstrated to be the primary site of infection with other strains of FMDV after aerosol [9] or intra-nasopharyngeal inoculation [13]. Consistent with previous studies in cattle [9, 13], large quantities of mixed mononuclear cells with MHCII+ and CD11C+ phenotyopes were in close proximity to infected epithelial cells, but did not contain FMDV antigens. These cells were interpreted as intra-epithelial dendritic (Langerhans-like) cells and macrophages. At 48-96hpi, FMDV-WT was localized to cytokeratin-containing cells in vesicle-like cavitations within pulmonary parenchyma and epithelial regions of palatine tonsils. This finding demonstrated that even during the fulminant stage of systemic infection, FMDV still maintained a restricted tropism, with highly selective preference for epithelial cells. The only exception to epithelial tropism was the very rare finding of small quantities of FMDV antigens localized to few CD11c + cells within lymphoid follicles of nasopharyngeal MALT during the viremic phase.

At the time points at which FMDV-Mut was examined in tissues, the microscopic distribution was nearly indistinguishable from FMDV-WT. The attenuated virus was localized exclusively to nasopharyngeal epithelium at 24hpi and pulmonary epithelium at 48-72hpi. Thus the selective tropism of FMDV was preserved in the mutant virus. Although infected cattle’s tissue morphology and phenotypic properties were similar between the viruses, there were substantially fewer detectable foci of infection with FMDV-Mut compared to FMDV-WT. Previous work has demonstrated significant differences in the innate immune response at these tissues at the macroscopic (whole tissue macerate) level [2]. Specifically, FMDV-WT was demonstrated to induce substantially more interferon type I/III activity than FMDV-Mut. On this basis, it is unlikely that the attenuated phenotype of FMDV-Mut was due to an enhanced innate immune response, since the tissues infected with FMDV-WT had substantially more interferon type I/III activity [2]. Additionally, the current study’s microscopic findings indicate that no morphologic or phenotypic evidence of enhanced immune response was identified to explain the attenuated phenotype of FMDV-Mut.

The pathogenesis of attenuated FMDVs have been investigated by several researchers. Although various aspects of virus-host interactions have been characterized, no study has ever provided the extent of detail of temporo-anatomic mapping of infection as is described in the current work. The striking in vivo similarities described herein between the virulent and attenuated viruses suggest that both viruses infect and propagate by similar mechanisms as is consistent with mechanistic similarities previously described in vitro [3]. Yet, the virulent virus replicated rapidly and caused systemic disease, whereas the attenuated virus replicated at a slower rate, as indicated by lower virus yields in tissues and secretions, and was ultimately arrested at the mucosal surfaces of primary infection. We propose that at the slower rate of replication, the host immune system was able to contain FMDV-Mut, even with a weaker immune response compared to that induced by FMDV-WT [2]. However, the current study cannot rule out the possibility of still-undetected mechanistic differences between the two viruses, or the host responses that they generated. Further investigation of other attenuated FMDVs may elucidate the detailed mechanisms associated with this disparity and whether this is a common theme of viral attenuation.

## Conclusion

The attenuated FMDV-Mut strain was shown to achieve all the early events of infection of cattle as occurred with the parental FMDV-WT. This included similar microscopic localization of both viruses within nasopharyngeal and pulmonary epithelial cells. The distinguishing features of infection with the virulent FMDV-WT were higher viral loads in tissues and secretions, establishment of viremia, and manifestation of typical clinical signs of FMD. Although the full complement of pathogenesis mechanisms of the two viruses remains incompletely elucidated, the current findings and previous works suggest that the impaired replication of the mutant is more responsible for attenuation than differences in host factors.

Additionally, the fine detail of tissue-specific virus loads reported herein provides previously unavailable extent of granularity to support within-host modeling of FMD in cattle.

## Abbreviations

BHK: Baby hamster kidney
BSL-3Ag: Biosecurity level 3 Agriculture
CD11c: Cluster of differentiation 11c
CPE: Cytopathic effect
Ct value: Cycle threshold value
FAE: Follicle-associated epithelium
FMD: Foot-and-mouth disease
FMDV: Foot-and-mouth disease virus
FMDV-Mut: Foot-and-mouth disease virus mutant
FMDV-WT: Foot-and-mouth disease virus wild type
GCN: Genome copy number
Hpa: Hours post aerosolization
IHC: Immunohistochemistry
Lpro: Leader proteinase
MALT: Mucosa-associated lymphoid tissue
MHCII: Major histocompatibility complex type II
MIF: Multichannel immunofluorescence
qRT-PCR: Quantitative reverse transcription polymerase chain reaction
RNA: Ribonucleic acid
TCID_50_: 50% Tissue culture infectious dose
VI: Virus isolation
vRNA: Viral ribonucleic acid
